# The Bologna criteria for poor ovarian response: a contemporary critical appraisal

**DOI:** 10.1186/s13048-015-0204-9

**Published:** 2015-11-17

**Authors:** Johnny S. Younis, Moshe Ben-Ami, Izhar Ben-Shlomo

**Affiliations:** Reproductive Medicine Unit, Department of Obstetrics and Gynecology, Poriya Medical Center, Tiberias, 15208 Israel; Faculty of Medicine, Bar-Ilan University, Galilee, Israel

**Keywords:** Poor ovarian response, Bologna criteria, Low ovarian reserve, Ovarian ageing, ART

## Abstract

Postponement of child bearing and maternal age at first pregnancy are on the rise, contributing considerably to an increase in age-related infertility and the demand for assisted reproductive technologies (ART) treatment. This brings to the infertility clinics many women with low ovarian reserve and poor ovarian response (POR) to conventional stimulation. The Bologna criteria were released to standardize the definition of POR and pave the way for the formulation of evidence-based, efficient modalities of treatment for women undergoing IVF-ET. More than four years have passed since the introduction of these criteria and the debate is still ongoing whether a revision is due. Women with POR comprise several sub-groups with diverse baseline distinctiveness, a major issue that has fueled the discussion. Although antral follicle count (AFC) and anti-Müllerian hormone (AMH), are considered good predictors of ovarian reserve, their threshold values are still not universally standardized. Different definitions for sonographic AFC and diverse assays for AMH are held responsible for this delay in standardization. Adding established risk factors to the criteria will lead to more reliable and reproducible definition of a POR, especially in young women. The original criteria did not address the issue of oocyte quality, and the addition of risk factors may yield specific associations with quality vs. quantity. Patient’s age is the best available criterion, although limited, to predict live-birth and presumably oocyte quality. High scale studies to validate these criteria are still missing while recent evidence raises concern regarding over diagnosis.

## Background

Maternal age at first pregnancy and age-related infertility are steadily increasing and the consequent demand for assisted reproductive technologies (ART) is on the rise [1–3]. Postponement of child bearing is common in the industrialized countries. This brings to the infertility clinics many women who are beyond the optimal age for conception. Hence, many are presenting with a diminished response to the standard stimulation protocols. The European Society of Human Reproduction and Embryology (ESHRE) published the Bologna criteria in 2011 in order to standardize the definition of poor ovarian response (POR) in a simple and reproducible manner [4]. Till that time the variability in the definition of POR has been striking [5]. The main purpose of the uniform criteria was to develop evidence-based efficient and appropriate protocols or modalities of treatment for such women undergoing IVF-ET treatment. An agreement was reached on the minimal criteria needed to define POR. At least two of the following three criteria had to be present to establish the definition: (1) Advanced maternal age (>40 years) or any other risk factor for POR. (2) A previous POR (≤3 oocytes with a conventional stimulation protocol). (3) An abnormal ovarian reserve test [i.e. antral follicle count (AFC) less than 5–7 follicles or anti-Müllerian hormone (AMH) below 0.5–1.1 ng/ml]. Since 2011 a significant body of discussion and debate has been published regarding needed revisions [6–10]. These points of discussion and concerns are summarized in Table 1.

**Table 1:** Main points of debate and concerns regarding the Bologna criteria for poor ovarian response

1. Homogeneity of population	
2. Cut-off values for	
a. Age	
b. Number of retrieved oocytes	
c. AFC and AMH	
3. Risk factors other than age	
4. Oocyte quantity versus quality	
5. Over diagnosis	
6. Large scale validation	

The purpose of the present review is to summarize this discussion and expound several points of the debate. The cut-off points for age and number of retrieved oocytes, included in the first two criteria, have been recently discussed [10]. In this review, a contemporary appraisal of the literature is carried out, concerning AFC and AMH threshold values, oocyte quantity versus quality, concern regarding over diagnosis and presenting evidence to include established risk factors into the criteria.

## Review

### POR includes several sub-populations

Poor ovarian response is accepted as a manifestation of low ovarian reserve and early ovarian aging. The primary theory being held to explain this process is the depletion of the ovarian pool of non-growing follicles, including primordial, intermediate and primary follicles, believed to be at its maximum in-utero and shrinking gradually towards menopause. Several mechanisms have been suggested to explain the decline in oocyte quantity and quality. These include possible differences in germ cell formation during fetal life, changes in the quality of the granulosa cells surrounding the oocyte as well as accumulated damage to the oocytes during childhood and reproductive life [11]. However, the exact mechanism(s) are still mostly obscure. Thus, any of several possible mechanisms may underlie POR in different patients and each suggested treatment mode may have different impact on oocyte quality and some may not improve the outcome [6].

In this regard, Papathanasiou maintains that women grouped under the Bologna criteria comprise several sub-populations with diverse baseline characteristics and unknown clinical prognosis [8]. For research purposes this posits potential bias if women from each sub-population are not recognized and accordingly evenly allocated between comparison groups. In response to his suggested stratified randomization by eight sub-populations, Venetis claims that stratification has several shortcomings and should be used only when there is clear evidence supporting such a design [9]. It is premature at this stage to perform stratified randomization when there is insufficient data to support such practice in RCTs on women conforming to the Bologna criteria.

Only ten years ago the original consensus criteria for the diagnosis of polycystic ovary syndrome (PCOS) were released [12]. PCOS is a relatively common condition believed to be caused by several mechanisms, which need to be clarified and explored. Moreover, it includes several phenotypes and sub-populations. Yet, this has not prevented the introduction of simple, clearly defined and reproducible criteria that paved the way for well-designed controlled studies, which examined different treatment modalities. Admittedly, when these criteria for PCOS were first introduced, concerns over disadvantages of these criteria, specifically related to various phenotypes and different sub-groups, have been also raised and discussed [13, 14]. However, these criticisms did not prevent the implementation of properly designed trials with good external validity that evolved into clear consensus on infertility treatment and various aspects of women’s health related to PCOS patients [15, 16].

Recently, few retrospective studies have evaluated the Bologna criteria to determine whether they may effectively identify infertile populations with low ovarian reserve and diminished chance of success undergoing ART treatment [17–21]. The live birth rate following conventional stimulation was shown to be similarly low, 6–7 % per started cycle, in three unrelated studies [18, 19, 21], supporting the validity of the Bologna criteria for POR, an encouraging step toward the adoption of a uniform definition. Further large scale studies are needed to validate these results.

### AFC and AMH threshold values

Antral follicle count and AMH are considered today the most informative biomarkers of ovarian reserve [22]. Their improved performance is the result of significantly stronger correlation with non-growing follicles and primordial follicle counts [22]. Although it is still debatable which of the two biomarkers is superior for ovarian reserve appraisal, both predict poor response and cycle cancellation as well as excessive response and ovarian hyperstimulation syndrome development with equivalent levels of accuracy and clinical value [23, 24]. Similarly, they also predict age at natural menopause, which is a related correlate of ovarian reserve [25–27]. Both AFC and AMH have also been tested for their potential to optimize treatment strategies for improving pregnancy outcomes in ART, and yielded encouraging results [28–30]. For those reasons they were appropriately chosen to serve as the third criterion of the Bologna definition of POR.

Nevertheless, both biomarkers still lack total consistency, thus flexible cut-off levels of AFC and AMH to predict POR were included in the Bologna criteria. For AFC, the cut-off values chosen ranged from less than 5 to less than 7, whereas for AMH the values were in the range from less than 0.5 to less than 1.1 ng/mL. Several methodological issues have been raised regarding sonographic antral follicle definition (2–5 or 2–10 mm) and different AMH assays (Immunotech–Beckman Coulter and Diagnostic System Laboratories) [31]. More challenging is the fact that the diagnostic performance of any biomarker of ovarian reserve, including these two, is methodologically dependent on the prevalence of POR in the studied population. The latter could affect the sensitivity and specificity of either test to detect POR, in turn dictating the chosen threshold. Obviously, patient selection is crucial when studying POR occurrence during ART treatment. Liberal policy of ART employment in POR women, such as in Israel may diverge from its strict use [32]. These factors should be well evaluated in prospective targeted future studies with appropriate external validation in order to reach clearly defined and reproducible threshold criteria for these biomarkers.

### Oocyte quantity versus quality

The original Bologna criteria did not address the issue of oocyte quantity versus quality, and this issue remains to be resolved. Chronological age has been considered as the best criterion available, although limited, to predict pregnancy and presumably oocyte quality. Until few years ago, none of the available ovarian reserve tests, including AFC and AMH were shown to predict pregnancy or live birth with sufficient accuracy [33, 34]. Moreover, the addition of these tests appears to add no value to chronological age [22].

In the last few years, well performed studies have shown a significant association between AMH and live birth prediction in ART, independent of age [35, 36]. A large recent review and meta-analysis concluded that it is probable that AMH has an association with oocyte quality independent of a woman’s age, but this association is likely weak to moderate at best. [37] This may be helpful when counseling couples before undergoing fertility treatment. However, its predictive accuracy is poor, especially when there is evidence to indicate that live births are possible with very low AMH levels opposing the use of AMH values alone to withhold treatment [38].

It should be noted that the contradicting findings of studies showing an association between AMH and live-birth and others negating such an association could be attributed to differences in AMH testing, study design and patient selection. Until there is an accurate biomarker that could examine oocyte quality and not quantity, the task of finding suitable criteria to predict live-birth would be intricate to accomplish.

The increasing miscarriage rate with advancing female age has been attributed to a decline in oocyte quality. A poor response to ovarian hyperstimulation is often an expression of a decrease in oocyte quantity. Although oocyte quality and quantity both decrease as a result of ovarian ageing, it is unclear to what extent these two processes are correlated to each other. Miscarriage rate following IVF-ET treatment would be an indirect measure of oocyte quality. Interestingly, Among women <36 years, miscarriage rates between poor and normal responders did not differ, whereas among women ≥ 36 years poor responders had a significantly increased miscarriage rate compared to normal responders [39]. These findings show that women with POR at young age likely have better oocyte quality as compared to more advanced age women with comparable POR. Even more, it emphasizes the importance of risk factors inclusion into the definition criteria.

### Concern regarding over diagnosis

According to the Bologna criteria one stimulated cycle is essential to fulfill the second criteria for the diagnosis of POR. However, patients over 40 years of age or with a risk factor for POR and an abnormal AFC or AMH value are classified as “expected POR” before engaging into treatment. “Expected POR” women, also described as diminished/low ovarian reserve cases, are usually urged to go forward for ART without hold at other modalities of treatment to maximize their suboptimal success rate.

Recent large scale studies have shown that the diagnosis of diminished/low ovarian reserve as an indication for ART treatment has increased significantly in the last few years [40, 41]. This increase has been shown to over-ride the expected natural raise of average age of mother at first birth. These results imply that diminished/low ovarian reserve diagnosis represents over diagnosis rather than improved detection [41] and the addition of diagnostic testing modalities, such as AFC and AMH, may has contributed to this increase.

What may concern more is the recently reported wide discrepancy in live-birth rate between women undergoing ART treatment with diminished/low ovarian reserve or “expected POR” and those with actual POR. While the live birth rate was reported to be optimistic in “expected POR” cases (17–24 % per cycle) [20, 42], it was shown to be particularly low in actual POR patients (6–7 % per cycle) [17–19, 21].

Taken together, the diagnosis of diminished/low ovarian reserve or “expected POR” as an indication for ART treatment is increasing, while their live birth rate is too optimistic, questioning the reliability of the diagnosis and suggesting over diagnosis. Careful and accurate employment of the Bologna criteria for POR and “expected POR” diagnosis are crucial to universally adopt and implement these criteria into the daily practice. Furthermore, the inclusion of risk factors into the first criteria seems to add clarity and reliability of POR definition.

### Risk factors of poor ovarian response

In order to reach a common and universal definition of POR, each one of the three criteria selected in the Bologna criteria should be simple, clearly defined and reproducible. Whereas maternal age (≥40 years), previous POR (≤3 three oocytes with conventional stimulation) and abnormal ORT (AFC < 5–7 or AMH < 0.5–1.1 ng/ml) were all fittingly defined in the Bologna criteria, the risk factors were not [4]. Specific risk factors unequivocally associated with POR were not defined. A want of well-established risk factors was voiced by us and adopted later by the ESHRE working group [7, 42].

The recommended list of risk factors for POR in young women below the age of 40 years was produced following a comprehensive search of the literature [43]. The list includes medical risk factors as short menstrual cycle length, single ovary, previous ovarian cystectomy, chronic smoking, unexplained infertility, previous chemotherapy and/or radiotherapy treatment. It also includes genetic risk factors such as family history of premature menopause, X chromosome derangements and fragile X mental retardation 1 (FMR1) pre-mutation.

In this review, an emphasis on recent studies, in particular since the release of the Bologna criteria, has been made. A special attention was made to examine contemporary evidence evaluating these risk factors in relation to ovarian response to hormonal stimulation, ovarian reserve tests, premature ovarian failure (POF) and early age at menopause as a proxy of accelerated ovarian ageing, representing different facets of ovarian reserve appraisal.

### Short menstrual cycle

Shortening of the menstrual cycle heralds the menopausal transition and is associated with inferior IVF results [44, 45]. Short menstrual cycle was attributed to low inhibin-B and high FSH levels accelerating follicular recruitment, dominance and ovulation.

In a prospective observational study Brodin et al. [46] evaluated a total of 6271 IVF/ICSI treatment cycles, and recorded that increasing age was associated with a subtle shortening of menstrual cycles. Menstrual cycle length was significantly associated with ovarian response to gonadotropin stimulation, embryo quality, and clinical pregnancy and live birth rates, independently of age.

In a recent analysis of 2015 oocyte donation cycles resulting in 3427 embryo transfers, Vassena et al. found that the shorter the cycle length, the poorer were the response to ovarian stimulation in the donors and pregnancy rate in the receivers [47].

Taken together, menstrual cycle length seems to have a role in POR detection, in women undergoing ART treatment, affecting both oocyte quantity and quality.

### Single ovary

Unilateral oophorectomy and ovarian cystectomy have been both implicated in the decrease of ovarian reserve. Recently, in a cross-sectional analysis of the Japan Nurses’ Health Study data were analyzed for 24,152 pre- and postmenopausal women who were 40 years or older at the baseline survey [48]. Median age at menopause in women who had undergone unilateral oophorectomy was 1.2 years earlier than controls. Single ovary was also associated with early menopause before 45 years of age [OR = 3.94] and POF [OR = 3.32].

Similarly, in a cohort study of 23,580 Norwegian women who were included in the population-based HUNT2 Survey during the years 1995–1997, women who had undergone unilateral oophorectomy were significantly younger at menopause than women without unilateral oophorectomy [49]. The relative risk of menopause following unilateral oophorectomy was 1.27.

### Ovarian cystectomy

Resection of an ovarian cyst may inadvertently cause damage to the normal ovarian reserve. Coccia et al. [50] conducted a prospective longitudinal cohort study of 302 patients who underwent laparoscopy for endometriosis between March 1993 and November 2007. They recorded menopause in 43 women (14.3 %), of whom seven went into POF. Women who underwent bilateral cystectomy were five years younger at menopause than those who underwent a unilateral operation.

The impact of endometriomas’ resection on ovarian reserve as determined by serum AMH was a subject to a meta-analysis of eight studies [51]. Pooled analysis of 237 patients showed a decrease in serum AMH concentrations after ovarian cystectomy with weighted mean difference of –1.13 ng/mL. Although heterogeneity was high, the results of this analysis support a deleterious effect of the excision of endometriomas on ovarian reserve.

In a cohort analysis of 835 women with ovarian insufficiency, 75 patients underwent ovarian surgery before the onset of ovarian failure. Of those 75 patients, 66 underwent cystectomy, mostly for endometriomas [52]. The mean age of patients at the time of surgery was about 28 years, and the mean interval to the onset of ovarian failure was about 6 years. The patient’s age at the time of surgery and the elapsed time to the onset of ovarian failure were well-correlated, supporting the role of ovarian cystectomy as a risk factor for the development of ovarian insufficiency.

In a recent case–control study on women with POR aged under 40 years, IVF results and live birth rate were evaluated to examine whether they depend on the etiology of low ovarian reserve [53]. Clinical pregnancy rate and live birth rate were significantly impaired in POR caused by a previous cystectomy of an endometrioma as compared with POR of unknown etiology.

Taken together, resection of an endometriotic cyst(s) adversely affects ovarian reserve, supporting its inclusion as an independent risk factor for POR. Data presented support an association with both oocyte quantity and quality.

### Chronic smoking

Cigarette smoking is the most established and consistently observed risk factor for younger age at menopause with a clear dose-response association [54]. In a prospective cohort study of 3545 middle aged Australian women, a 21-year follow-up revealed that women who smoked cigarettes were more likely to experience earlier menopause than non-smokers. The impact of smoking on earlier age at menopause was independent of other variables suggesting a causal relationship [55]. Furthermore, in the largest population-based study of AMH, involving 2320 premenopausal women, current but not past smoking was associated with a lower age-specific AMH percentile [56].

During a 14-year follow-up of the Penn Ovarian Aging Study [57], of 401 women 39.2 % were smokers at the mean entry age of 41.5 years, AMH was a strong predictor of median time to menopause. Smoking significantly shortened the time to menopause. Combined, age and smoking were found as independent contributors to the predictions of AMH levels.

In an ART setting, Caserta et al. [58] found in a group of 102 smokers lower AFC and higher basal FSH level as compared to 194 non-smokers. Furthermore, the number of pack-years was negatively correlated to AFC and positively correlated to FSH levels. Studying 80 current active smokers with 197 non-smokers, Fréour et al. [59] found the same results regarding AMH and AFC and also that smokers also experienced poorer IVF outcome with decreased ovarian response, reduced top embryo proportion, lower pregnancy and live birth rates.

In another report, Fréour et al. [60] studied the morphokinetics of embryos after IVF-ICSI in 135 women, 23 smokers and 112 non-smokers. Time-lapse analysis showed that most cleavage events occurred significantly later in smokers than in nonsmokers, leading to poor cycle outcome in smokers.

There is but one report which does not support detrimental effect of smoking on the ovary and ART results [61], but it did not examine the effect of smoking with regards to patients with low ovarian reserve. Thus, smoking should stand as a discrete risk factor for POR.

Taken together, numerous studies support chronic smoking as a risk factor for POR, affecting both oocyte quantity and quality.

### Unexplained infertility

This factor is the least explored risk factor, among the 9 risk factors presented. In the last few years, to the best of our knowledge, no study has been performed to target the association between unexplained infertility and low ovarian reserve or POR.

In an earlier cohort study of 2392 women, analysis was performed using age at menopause as proxy for accelerated ovarian ageing. Several measures related to sub-fertility were analyzed [62]. All measures examined were significantly associated with early menopause supporting the notion that both are expressions of the same accelerated ovarian ageing.

Randolph et al. [63] conducted a prospective observational study in a small group of women rigorously defined as unexplained infertility. Their early follicular FSH was significantly higher compared to a matched control, pinpointing to diminished ovarian reserve.

In another study of infertile women undergoing hormonal stimulation for IVF, a prospective cohort of patients with unexplained and mild male factor sub-fertility was compared to a large retrospective cohort of women with unexplained, mild male and tubal sub-fertility [64]. In both cohorts, women with idiopathic or mild male sub-fertility showed POR while those with tubal factor did not.

Taken together, available studies support the notion that unexplained infertility is a risk factor for low ovarian reserve and POR.

### Previous chemotherapy and/or radiotherapy treatment

This is a well established risk factor for POR and early ovarian aging, leading to the recent advice to young women before chemotherapy or pelvic radiotherapy to consider novel fertility preservation measures before starting their medical treatment.

In a cross-sectional analysis of data 71 cancer survivors aged 15–39 years were compared to 67 same age healthy, unexposed women and to 69 regularly menstruating women of late-reproductive age (40–52 years) [65]. Early follicular ovarian reserve tests (AFC, AMH and FSH), were significantly inferior in exposed as compared to unexposed women. Alkylating agent dose score was significantly associated with increased levels of FSH and decreased levels of AMH. Exposure to pelvic radiation was also associated with impairment in FSH, AMH, AFC and ovarian volume. AMH was similar in women previously exposed to high-dose cancer therapy and 40–42 year old controls. All in all, among cancer survivors ovarian reserve tests, endocrine and sonographic, were impaired, in a dose-dependent manner, compared to unexposed women of similar age.

Krawczuk-Rybak et al. [66] studied 33 young female cancer survivors who were treated previously (6–11 years earlier), for a period above 5 years and compared them to 34 healthy controls. The group of survivors was divided according to the risk of gonadotoxicity into low (LR), median (MR) and high risk (HR) groups. Serum AMH level at baseline was significantly lower in the HR group compared to the control and the LR + MR groups. In the HR group AMH level continued to decrease and FSH to increase progressively after 5 years. In the LR + MR group, the levels of AMH and FSH were normal at baseline, but after 5 years AMH decreased and FSH increased indicating that chemotherapy during childhood and adolescence causes serious and progressive loss of ovarian reserve. High risk gonadotoxic treatment in this setting may thus lead to POF.

In a recent cross-sectional study 53 girls at median age 13.9 years who survived cancer were recruited at least 1 year from completion of cancer therapy and tested for ovarian reserve [67]. Thirty-one of the 53 patients (58 %) had AMH value <1 ng/mL and 17 (32 %) had FSH value of >12 IU/mL showing low ovarian reserve. Patients exposed to high-risk chemotherapy or pelvic irradiations were at significantly higher risk for early ovarian ageing. AMH level was also significantly lower in the patients who had delayed puberty emphasizing the deleterious impact of chemotherapy on ovarian reserve in childhood and adolescence.

### Family history of premature menopause

In a cross-sectional study, Bentzen et al. [68] obtained data on a prospective cohort of 863 health care workers aged 20–40 years who underwent ovarian reserve testing. Of these 527 disclosed their mothers’ age at natural menopause. A significant correlation was found between the mother’s age at natural menopause and daughters’ AFC and AMH values. The rate of decline in AFC and AMH was also associated with age at maternal menopause, supporting a genetic component in early menopause and low ovarian reserve.

In a recent study two groups were evaluated. The first consisted of 164 mother–daughter pairs. The second included 150 women, with regular menstrual cycles, in whom AMH and mother’s age at natural menopause were recorded prior to a 12-year follow-up period awaiting the appearance of natural menopause [26]. Both age and mother’s age at natural menopause were significantly associated with daughter’s time to menopause in the two cohorts. Multivariate analysis suggested a 47 % improvement in predictive accuracy of daughter’s age at natural menopause by adding AMH to the model of age and mother’s age at natural menopause.

Taken together, mother’s age at natural menopause seems to have a genetic role in predicting daughters’ ovarian reserve and therefore ought to be employed as a risk factor for POR.

### X chromosome derangements

This is a well established risk factor for POF, diminished ovarian reserve, POR and early menopause. Women with structural and numerical abnormalities of the X chromosome make up the largest subgroup with primary ovarian insufficiency. Reproductive disorders in women with Turner’s syndrome (45,X) arise from lack of all or part of the X chromosome. Although one X chromosome is sufficient to allow ovarian differentiation, oocytes need two active X chromosomes. In women with mosaic Turner’s syndrome (45,X/46,XX), menarche can take place and menstruation may continue for several years [69]. Other genetic derangements which could involve the X chromosome are monosomy, trisomy, inversion or translocation. Genetic mechanisms include reduced gene dosage and non-specific chromosome effect impairing meiosis, decreasing the pool of primordial follicles and increasing atresia due to apoptosis or failure of follicle maturation.

In a prospective case-control study, 18 women who experienced recurrent miscarriages and had mosaicism of X-chromosome aneuploidies were compared to two control groups, 20 women with a balanced structural autosomal rearrangement and 135 women without chromosomal abnormalities [70]. Women with X-chromosome mosaicism without a balanced autosomal structural rearrangement had a significantly higher incidence of POR, occurring in 44.4 % of cases as compared to 9.6 % of controls. In comparison with controls without chromosomal abnormalities, women with a balanced autosomal structural rearrangement also had higher incidence of POR, but the groups were too small for the difference to reach statistical significance.

The role of genes as determinants of ovarian aging receives growing attention in the last decade and the information is rapidly expanding. Methods which are used to elucidate the role of specific genes involved in ovarian aging include comparative genomic hybridization (CGH), array CGH (aCGH), genomewide linkage analysis, candidate gene-association studies, genome-wide association studies and transgenic animal models. This have been recently reviewed [71] and successfully employed in cases of idiopathic POF involving X chromosome to detect partial Xp duplication and Xq deletion in spite of a cytogenetically 46,XX normal karyotype [72–74].

### Fragile X mental retardation 1 pre-mutation

The number of CGG repeats in the X-linked gene known as fragile X mental retardation 1 (FMR1) can be responsible for various clinical conditions. Normal alleles have 26–34 repeats of the trinucleotide, whereas the full mutation has >200 repeats and is responsible for the clinical features of fragile X syndrome, which is the commonest cause of inherited mental retardation or genetically caused autism. Premutation alleles range from 55 to 199 CGG repeats and can cause either a neurological degenerative disorder in male carriers or premature ovarian failure in approximately 13 % of the female carriers. In addition, premutation alleles are unstable and can be expanded to full mutation over several generations [75, 76].

It has been reported that women carrying a premutation allele have a higher likelihood of experiencing menopause approximately 5 years earlier and having higher levels of FSH for any age over 30 years, compared with non-carriers. However, the relationship between the number of CGG counts and age of menopause is not linear and it appears that women with 80–100 repeats are at greater risk of POF than women with over 100 repeats [76].

In an IVF-PGD setting for fragile X syndrome analysis, 27 patients with the FMR1 mutation (5 with full mutation, 22 with premutation) undergoing 79 cycles were compared to 33 controls with other genetic diseases undergoing 108 treatment cycles [77]. FMR1 mutation carriers required significantly higher doses of gonadotropins, which nevertheless yielded significantly fewer numbers of oocytes, indicating diminished ovarian reserve in FMR1 carriers.

In a cohort of 535 infertile women < 42 years of age with low ovarian reserve, defined by elevated FSH or poor response to hormonal stimulation, participants were analyzed for FMR1 premutation (55–200 repeats) and intermediate alleles (45–54 repeats) and compared to 521 controls [78]. The frequencies of the premutation as well as intermediate alleles were significantly higher in women with low ovarian reserve compared to controls, 1.3 % vs 0.2 % for premutation and 3.2 % vs 0.2 %, for intermediate alleles.

It is still unclear whether the grey zone of intermediate alleles with up to 54 CGG counts has an association with low ovarian reserve and early ovarian aging. Although primary reports indicated an increased risk for the development of premature ovarian failure and impaired ovarian reserve tests [79–81], recent well controlled studies found no negative effect of intermediate-sized CGG repeats on ovarian stimulation and clinical outcome in either oocyte donation setting [82], development of premature ovarian failure [83] or age at natural menopause [84].

### The debate on risk factors inclusion

It seems that the nine situations discussed above have evidence to support their employment as risk factors for the development of POR in the ART setting. Three of these risk factors, including short menstrual cycle, endometriotic cystectomy and chronic smoking have evidence to support their association with both quantity as well as quality of the retrieved oocytes, affecting embryo quality and pregnancy achievement. Prospective targeted studies are encouraged to strengthen these associations. The other six factors have evidence to support their role in the quantity of oocytes and more studies are needed to examine their association with oocyte quality. Including risk factors in the Bologna criteria should yield specific associations between oocyte quantity and quality. Most of these factors are easy to detect by taking simple medical history, making their use easily applicable. Unexplained infertility seems to be the least explored risk factor for POR and future well designed studies are needed to further explore this association.

Since the release of the Bologna criteria few studies have evaluated their validity, following conventional stimulation in ART. The risk factors included were different among these studies [18–21]. While part of these studies did not note which risk factors were looked for [18, 21], one study included ovarian endometrioma, previous ovarian surgery, previous chemotherapy, genetic abnormalities and shortening of the menstrual cycle as risk factors [19] and another included only history of ovarian surgery or endometrioma [20]. These differences in risk factors inclusion demonstrate lack of clarity in the Bologna criteria for POR which may adversely affects their reproducibility and reliability. The need for standardization of risk factors seems to be essential.

It might be argued that any of these risk factors could not solely identify or predict young POR patients with high accuracy. However, adding a previous POR (second criterion) or a pathological ovarian reserve test (third criterion) to a proven risk factor would fulfill the Bologna criteria for POR. Similarly, previous POR (second criterion) or a pathological ovarian reserve test (third criterion) cannot solely predict POR with high accuracy. It was clearly cited in the Bologna original report on POR definition that one-third of previous poor responders will have a normal response in subsequent cycles [84]. Moreover, while AMH and AFC are the most reliable and accurate markers of ovarian reserve [85, 86], their overall performance in the prediction of poor response is less than optimal [33]. Most importantly, this was the original idea when employing the same logical approach for PCOS diagnostic criteria; each criterion used alone is insufficiently accurate to identify women with the highest probability of being a real POR, and more than one criterion should be contemporaneously present in each subject.

It is also implicit that some of the risk factors are well established, while others are still controversial and some novel candidates may be identified in the near future [7, 10], but this should not hold back the incorporation of the proven risk factors into the criteria. A second meeting of an expert working group on POR definition may be organized to discuss which risk factors have enough evidence to be included in the criteria.

Accurate definition of POR or “expected POR” in women below the age of 40 may contribute materially to a more appropriately tailored treatment approach. In addition, it may open new doors for the incorporation of young POR women into future targeted trials testing new strategies or modalities of treatment. Most importantly, it may enable health providers to recommend, at appropriate biological time, early pregnancy achievement or fertility preservation in women at risk.

## Conclusions

Poor ovarian response to conventional stimulation comprises several sub-populations of low ovarian reserve with diverse baseline characteristics. This should not preclude steps to adopt a uniform definition of POR, one of the main challenges of modern reproductive medicine. The Bologna criteria for the definition of POR have introduced a key step forward. However, the discussion is still ongoing regarding age, number of retrieved oocytes as well as AFC and AMH cut-off levels, adopted by the ESHRE consensus criteria. Concern has also been raised regarding absent association with oocyte quality and presumably pregnancy achievement as well as over diagnosis when adopting these criteria. Large scale validation studies are still lacking to universally adopt the Bologna POR criteria. Time has come to include established risk factors into the criteria. This will lead to a more simple, reproducible and reliable definition of POR. Moreover, the addition of risk factors for POR, especially in young women, may avoid confusion, yield specific associations with oocyte quality versus quantity and prevent over diagnosis.

## Abbreviations

ART: Assisted reproductive technologies
AFC: Antral follicle count
AMH: Anti-Müllerian-hormone
POR: Poor ovarian response
PCOS: Polycystic ovary syndrome
FMR1: Fragile X mental retardation 1
POF: Premature ovarian failure
